# Seroprevalence of *Toxoplasma gondii* in Wild Rats (*Rattus rattus*) in Northern Iran

**DOI:** 10.1155/2021/6655696

**Published:** 2021-05-27

**Authors:** Seyed Abdollah Hosseini, Saeid Abediankenari, Afsaneh Amouei, Shahabeddin Sarvi, Mehdi Sharif, Fatemeh Rezaei, Ahmad Daryani

**Affiliations:** ^1^Toxoplasmosis Research Center, Mazandaran University of Medical Sciences, Mazandaran, Sari, Iran; ^2^Department of Parasitology, School of Medicine, Mazandaran University of Medical Science, Mazandaran, Sari, Iran; ^3^Immunology Department, Faculty of Medicine, Mazandaran University of Medical Sciences, Sari, Iran; ^4^Immunogenetics Research Center, Mazandaran University of Medical Sciences, Sari, Iran; ^5^Department of Parasitology, School of Medicine, Sari Branch, Islamic AZAD University, Sari, Iran

## Abstract

Rodents are considered as reservoir hosts for various pathogens (such as *Toxoplasma gondii*) and have been revealed to play an important role in the spread of several infectious diseases to humans and other animals. The aim of this investigation was to survey the prevalence of anti-*T. gondii* IgG antibodies in wild rats in Northern Iran. One hundred rats were caught using rat traps set in different areas in Northern Iran (September 2017). The thoracic cavity of each rat was opened, and then the blood sample was collected from the heart. IgG anti-*Toxoplasma gondii* antibodies were detected using the modified agglutination test (MAT) with a cutoff value equal to 1 : 40. Overall, 56% of rats were infected by *T. gondii*. Considering the sex of rats, 45% of male and 55% of female rats were seropositive, but the differences were not statistically significant. There was a significant difference between seropositivity and habitat types and age of rodents. Our findings have public health implications and confirm the high seroprevalence of *T. gondii* infection in northern Iran. The study established that wild rats represent an important and persistent wildlife intermediate host reservoir for *T. gondii*.

## 1. Introduction

*Toxoplasma gondii (T. gondii)*, a unicellular protozoa, is a common parasite among animals and humans [1]. It is responsible for one of the most widespread zoonotic protozoan infections that is usually asymptomatic in healthy individuals but may cause serious complications in immunocompromised hosts [2].

*T. gondii* infection is usually transmitted to humans via the consumption of sporulated oocyst-contaminated food or consumption of undercooked meat containing cysts, and vertical transmission is from the mother to the fetus via the placenta [3].

Cats are the definitive hosts for *T. gondii*. Rodents serve as reservoir hosts for various pathogens and have been shown to play a chief role in the transmission of several infectious diseases to animals and humans [4, 5]. Rats as herbivores are susceptible to *T. gondii* infection due to the consumption of food/water contaminated with oocysts present in environments [4, 6]. The investigation of the seroprevalence of the parasite in rats could be of epidemiological implications for the reason that rats can serve as sources of *T. gondii* tissue cysts for the cats [6].

In Northern Iran, so far, several studies have been conducted on the prevalence of *T. gondii* in humans and different animals, but there are no data about the seroprevalence of *T. gondii* in rodents. This study aimed to survey the seroprevalence of this protozoan in wild rats in Mazandaran Province, Northern Iran.

## 2. Materials and Methods

### 2.1. Study Population

In this descriptive investigation, 100 rats (*Rattus rattus*) were caught using rat traps set in different sites in Sari city, Iran (September 2017). Gender (male and female), weight (adult rats 200 g), and habitat type (urban and rural) were recorded. Because older rats are generally heavier, age was estimated based on body weight [7]. The animals were transported to the Parasitology and Mycology Laboratory at the Mazandaran University of Medical Sciences. Then, rats were anesthetized and exterminated with ether, then the thoracic cavity of each rat was opened, and the blood sample was taken from the heart for immunodiagnosis.

### 2.2. Preparation of the MAT Antigen

Adult female Swiss albino mice were procured from Animal Research Center, Mazandaran University of Medical Sciences. Approximately 3 × 10^4^ tachyzoites of the RH strain of *Toxoplasma gondii* were inoculated into the mice. Five days after the infection, the animals were euthanized by ether, and the thoracic cavity was opened. By using a syringe, the fluid was aspirated and diluted with sterile phosphate-buffered saline (PBS, pH 7.2) containing penicillin and streptomycin. After the centrifugation, tachyzoites were suspended in 2% formaldehyde solution and kept overnight. The sediment was washed in PBS three times to remove formaldehyde. Alkaline antigen buffer was arranged by dissolving 3.5 g of sodium chloride, 1.54 g of boric acid, and 2 g of bovine serum albumin in 450 ml deionized water. At that time, 12 ml of 1 N NaOH was added, and pH of the solution was adjusted to 8.95. The volume of the solution was made up to 500 ml and was stored at 25°*C*. Formalin-killed antigen was diluted with alkaline buffer consequently to make up the final concentration of 1 × 10^7^ tachyzoites/ml.

### 2.3. Preparation of Serum

The serum samples were diluted with sterile PBS (1x, pH 7.2) including 4 g sodium chloride, 0.1 g potassium chloride, 0.57 g disodium hydrogen orthophosphate dihydrate, 0.1 g potassium dihydrogen orthophosphate, and 500 ml distilled water. The initial dilution was started at 1 : 10 to 1 : 800.

### 2.4. Performance of the Modified Agglutination Test (MAT)

Fifty *μ*l of the antigen combination was added to each well (U-bottom plate), then it was mixed, and 50 *μ*l of sera was added. Finally, twenty microliters of 0.2 M (14 ml/liter) 2-ME diluted in PBS was distributed in each well, and after mixing, the plates were incubated at 22°C overnight.

After 24 hours, the plates were read on a black background with lateral light. A negative reaction exhibited a central discrete opaque dot or button of the well, whereas diffuse opacity across the entire diameter was recorded as positive. In this study, it adopted a cutoff value equal to 1 : 40 for rat sera by MAT.

### 2.5. Statistical Analysis

The results were analyzed using SPSS—18.0 software package (SPSS Inc., Chicago, IL, USA). *P* value less than 0.05 was considered statistically significant. Chi-squared test was used to compare the difference between the seroprevalence of *T. gondii* and the variables such as age, sex, and habitat type.

## 3. Results

In this cross-sectional study, anti-*T. gondii* IgG antibodies were found in 56 rats' sera (56%) by the MAT method. From 56 positive sera, 2 samples were positive in MAT titers of 40, 34 samples in 80, 14 samples in 160, 5 samples in 320, and 1 sample in 640.

Considering the gender of rats, 42 rats were male and 58 rats were female. The MAT result showed that 45% of male and 55% of female rats were seropositive for *T. gondii*, but the differences were not statistically significant (*P*=0.83).

Based on the habitat type of rats, 76 rats were in rural and 24 rats were in urban. The serological result showed that 61.8% of seropositive rats were in rural and 34.6% were in urban. The statistical analysis showed that there was a significant difference between seropositivity and habitat types of the rodents (*P*=0.028).

Moreover, in this investigation of 100 rats, 22 rats were juvenile, and 78 rats were adults. The serological finding showed that 36.3% of seropositive rats were juvenile and 61.5% were adults. The statistical analysis indicated a significant difference between the age of animals and seropositivity to *T. gondii* infection (*P*=0.035) (Table 1).

## 4. Discussion

The present study is the first investigation on the seroprevalence of *T. gondii* in the rats using the MAT method in Northern Iran. According to a literature review, numerous laboratory techniques have been established to identify the seroprevalence of *T. gondii* in the serum of infected rats, for example, enzyme-linked immunosorbent assay [8], latex agglutination test, indirect hemagglutination antibody, Sabin–Feldman dye test, and indirect immunofluorescent antibody test. While these ways seem to be sensitive and specific, the methods are costly and require to be accomplished in specific laboratories [9]. MAT is a simple and sensitive method for the diagnosis of anti-*T. gondii* IgG antibodies in humans and especially in animals [10]. Therefore, we used this method to define the prevalence rate of *T. gondii* in the present study.

Our results showed that the seroprevalence of the infection in rats in northern Iran was 56%. The high seroprevalence of this unicellular parasite in the wild rats in this area is very important because the infected rats can easily transmit *Toxoplasma* to other animals.

The wild rats are considered as a reservoir of *T. gondii* tissue cysts for cats, dogs, and other animals [11]. On the contrary, the incidence of *T. gondii* infection in cats actually reflects the infection rate in local rodents because cats are thought to be infected by eating these infected rats.

Regardless of the type of serological procedure, the seroprevalence rate of *T. gondii* infection in rodents in the world is variable [11, 12], for example, 0.3% in the USA [13], 0.8% in the West Indies [14], 3.2% in southern China [12], 23.4% in Panama [11], 35% in the UK [15], and 58% in the Philippines [16].

There are few studies on seroprevalence rates of *T. gondii* in rats in Iran. Mosallanejad et al. evaluated the rate of *Toxoplasma* infection in 127 serum samples from wild rats in southwestern Iran (Ahvaz) using the immunochromatographic assay to detect IgG anti-*T. gondii* antibodies. Thirty-one (24.41%) showed antibodies against *T. gondii* [17].

The prevalence rate of *T. gondii* in our study is similar to the study conducted in the Philippines by Salibay et al. and higher than other studies [16, 18, 19]. In Northern Iran, seroprevalence of *T. gondii* infection in different groups of humans and animals is very high as the seroprevalence of *T. gondii* was 74.6% in females referred to health laboratories before marriage [20], 75.6% in immunodeficiency patients [21], 58.8% in pregnant women [22], 35% in sheep, 30% in goats [23], and 40% in cats [24].

In Northern Iran, the high seroprevalence of *T. gondii* infection could be due to the following factors: humid and temperate climate; absence of routine treatment for the infection in animals; and a considerable abundance of cats and other animals. In this area, high moisture (almost 90%) and environmental temperature (16–22°C) provide favorable conditions for the sporulation and survival of *T. gondii* oocysts of [25].

The age of hosts is the main factor in the prevalence of *T. gondii* infection, and usually, older animals are highly infected than younger ones; it is estimated that, with age, the risk of exposure to the parasitic infection increases significantly [18, 26]. Our analysis showed that seroprevalence of the infection in adult rats is higher than juvenile rats (61.5% to 36.3%). There was a significant difference among the seroprevalence rate of *Toxoplasma* and age (*P*=0.035).

In our study, 61.8% of rats living in rural environments were infected with *T. gondii* compared to 34.6% of rats captured in urban areas. There was a significant difference among the seroprevalence of *T. gondii* in rats and the living place (*P*=0.028). These findings are similar to most studies and dissimilar to the findings of studies conducted by Dabritz et al. in California, USA [27]. In rural areas, abundance of cats is an important factor in the spread of *T. gondii* infection. Cats are a chief source of the *Toxoplasma* infection [28]. The definitive host excretes many oocysts of *T. gondii*. The sporulated oocysts of *T. gondii* can survive for numerous months in moist environments [27].

Due to the richness of oocysts in the environment, predatory animals may become infected with *T. gondii* by ingesting these oocysts that can lead to an increase in the infection among the cats. Moreover, rats are infected by ingesting oocysts excreted by cats [29].

In the current survey, there was no significant difference in the prevalence of *Toxoplasma* among male and female rats. However, prevalence of this parasite in females (56.8%) was higher than in males (54.7%). These results are comparable to the study conducted by Yin et al. in China [12] and in contrast to the results of the study conducted by Cabanacan-Salibay and Claveria in the Philippines [30].

The rat-cat cycle plays a major role in the transmission of *T. gondii* infection to humans [26]. Cats, as the most important host of *T. gondii*, can become infected with *Toxoplasma* via digestion of infectious oocysts in the environment or via the consumption of tissue cysts present in intermediate hosts including rats [29]. Also, this definitive host usually excretes oocysts in its feces after the initial infection with *T. gondii*, which causes the spread of *T. gondii* infection [26].

There are many factors that have an influence on the epidemiology of *T. gondii* infection, such as the density of cats and intermediate hosts such as rats, environmental conditions (temperature and humidity), geographical area, and food processing [25]. Reducing the population of feral cats, spaying cats, collecting house cat feces in litter boxes, complete cooking of all types of meat, promoting severe hand washing, and decreasing the records of rats are significant in the control of the infection [31].

This study is the first investigation of *T. gondii* infection in wild rats in Mazandaran Province, Northern Iran. Our findings have public health implications. Also, they confirm the high prevalence of *T. gondii* infection in Northern Iran. The investigation determined that wild rats are an important and insistent wildlife intermediate host reservoir for the infection in northern Iran. In general, the seroprevalence of *Toxoplasma* infection in wild rats may be lower than other animals, but the population of rats is higher than other animals. Therefore, infected wild rats are a chief source in the transmission of *Toxoplasma* infection in animals, and therefore, integrated strategies and procedures should be taken to control the infection in wild rats. It seems further studies should be conducted on domestic and wild animals to determine the presence of host reservoirs of *Toxoplasma* in this area.

## Figures and Tables

**Table 1:** Statistical analysis of *T. gondii* seropositivity in wild rats in Northern Iran based on gender, age, and habitat type.

Characteristics	No.	No. of positive samples with MAT titers					No. of pos.	% of pos.	95% CI	*P* value
		40	80	160	320	640				
Gender										
Male	42	1	17	4	1	0	23	54.7	38.6-70.1	
Female	58	1	17	10	4	1	33	56.8	43.2-69.8	0.83
Total	100	2	34	14	5	1	56	56		

Age										
Juvenile	22	0	6	1	1	0	8	36.3	24.3-67.7	
Adult	78	4	32	8	3	1	48	61.5	47.2-69.9	0.035 ^*∗*^
Total	100	4	38	9	4	1	56	56		

Habitat type										
Rural	76	1	29	11	4	2	47	61.8	49.9-72.7	
Urban	24	1	6	1	1	0	9	34.6	17.2-55.6	0.028 ^*∗*^
Total	100	2	35	12	5	2	56	56		

^*∗*^Significant difference.

## Data Availability

The data used to support the findings of this study are available from the corresponding author upon request.
